# La synovite villonodulaire de la cheville, une localisation rare: à propos d'un cas

**DOI:** 10.11604/pamj.2016.23.90.8838

**Published:** 2016-03-15

**Authors:** Abdellatif Benabbouha, Jonathan Basinga, Ismail Anteri, Abdelouab Jaafar

**Affiliations:** 1 Service de Chirurgie Orthopédique et Traumatologique I, Hôpital Militaire d'Instruction Mohamed V, Rabat, Maroc

**Keywords:** Villonodular synovitis, ankle, synovectomy, Villonodular synovitis, ankle, synovectomy

## Abstract

La synovite villonodulaire (SVN) est une prolifération pseudotumorale bénigne rare de la synoviale articulaire, d’étiologie inconnue. Elle peut aussi se développer au sein des bourses séreuses, des gaines tendineuses. Généralement, elle atteint les grosses articulations notamment le genou et la hanche. La localisation de la cheville est rare, avec seulement quelques cas publiés dans la littérature. Nous rapportons un cas de patiente de 52 ans présentant une SVN de la cheville droite. Elle a bénéficié d'une synovectomie subtotale. A deux ans de recul, il n'y avait pas de récidive clinique.

## Abstract (en)

Pigmented villonodular synovitis (PVNS) is a rare pseudotumoral proliferative benign condition of unknown etiology, affecting synovial joints. It can also develop in the synovial bursae and in the tendon sheaths. It usually affects large joints, such as the knee and the hip. Ankle is a rare location, with only a few cases reported in the literature. We report a case of a 52 year old patient with a PVS of the right ankle. She received a subtotal synovectomy. After 2 years of follow-up there was no clinical recurrence.

## Introduction

La SVN est une affection proliférative rare appartenant à la dystrophie synoviale bénigne, caractérisée par une hyperplasie villeuse ou nodulaire de la synoviale, d’étiologie inconnue [1]. Cette pathologie est habituellement mono-articulaire, touchant les grosses articulations telles le genou, la hanche. La localisation de la cheville est très rare avec seulement quelques cas publiés dans la littérature. Nous rapportons un cas de SVN touchant l'articulation de la cheville.

## Patient et observation

**Figure 1:**
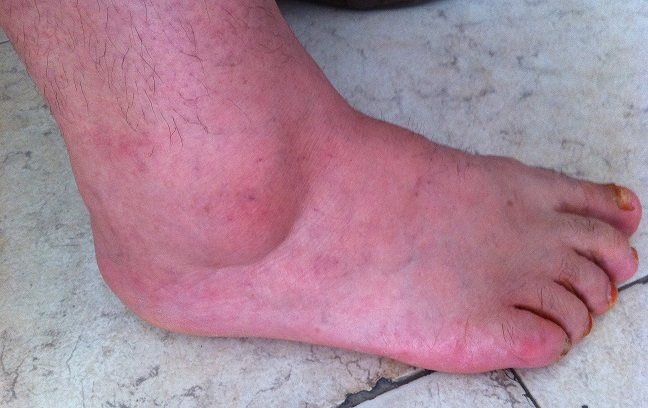
Tuméfaction prémalléolaire latérale

**Figure 2:**
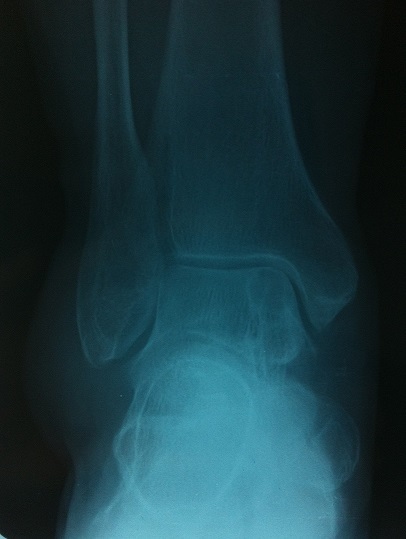
Radiographie de la cheville de face montrant une hyperdensité des parties molles

**Figure 3:**
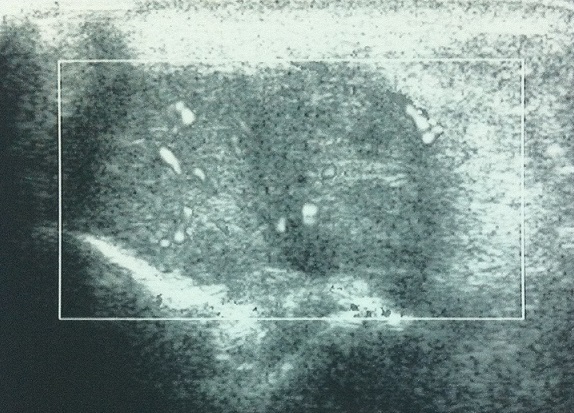
Échographie de la cheville objectivant une formation hypoéchogène

**Figure 4:**
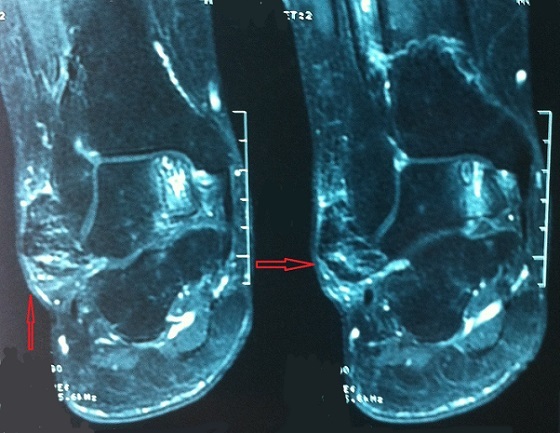
IRM de la cheville montrant une masse intra articulaire sous taliene hétérogène

Une femme de 52 ans avec des antécédents d'entorse à répétition de la cheville, qui présentait des douleurs mécaniques invalidantes de sa cheville droite d'installation progressive, évoluant depuis 20 mois, rebelles au traitement antalgique et une tuméfaction malléolaire externe qui augmentait progressivement de volume. Ce tableau évoluant dans un contexte d'apyrexie et de conservation de l’état général. L'examen clinique retrouvait une masse de consistance ferme, mesurant 3cm sur 2cm, peu sensible à la palpation et sans signes inflammatoires en regard (Figure 1). L'examen vasculo-nerveux était sans particularité. La radiographie conventionnelle de la cheville de face et de profil étaient normales en dehors d'une augmentation de la densité des parties molles (Figure 2). L’échographie révélait une formation hypoéchogène (Figure 3) avec un épanchement articulaire de moyenne abondance. L'imagerie par résonnance magnétique (IRM) montrait la présence d'une masse hétérogène intra-articulaire sous talienne envahissant la gouttière antérolatérale de cheville, se rehaussant après l'injection de produit de contraste faisant évoquer une pathologie pseudotumorale (Figure 4). On notait également un épaississement de la synoviale. La biopsie chirurgicale a été réalisée par un abord direct et l’étude anatomo-pathologique confirmait le diagnostic de SVN. La patiente a donc bénéficié d'une résection de la masse par voie antérolatérale avec une synovectomie subtotale. Aucun geste osseux n’était effectué. A deux ans de recul, il n'y avait pas de récidive clinique et la gêne fonctionnelle était modérée. Cependant une rechute locale ne peut être pas écartée, imposant une surveillance clinique et radiologique rapprochée.

## Discussion

Décrite pour la première fois par Chassignac puis par Jaffe et Al. en 1941 [2], la SVN est une tumeur fibre-histiocytaire responsable d'une hyperplasie des villosités synoviales pouvant confluer en nodule. Elle peutaussi toucher les gaines tendineuses et les bourses séreuses. Morphologiquement, nous distinguons des formes diffuses intéressant l'ensemble de la synoviale et des formes localisées. Toutes les articulations peuvent être concernées, mais les plus souvent touchées sont celles du genou (80%), de la hanche (10%), de la cheville (5%) et exceptionnellement l'articulation temporo-mandibulaire, et le rachis. Il s'agit d'une affection rare, Myers et Al. [3] estimaient son incidence à 1,8 cas par million d'habitants par an en 1980. Elle touche essentiellement l'adulte jeune âgé de 20 à 50 ans, sans déséquilibre de sexe, ce qui correspond à notre cas. L’étiopathogénie de la SVN est mal connue et encore discutée aujourd'hui. Bien que plusieurs hypothèses aient été émises telles que la survenue d'une prolifération synoviale liée à une inflammation chronique, unenéoplasie bénigne d’étiologie inconnue, une anomalie du métabolisme lipidique local et des traumatismes répétés [4]. Certaines aberrations cytogénétiques sont détectées dans la majorité des cas de SVN mais pas dans tous [5]. Certains auteurs comme Perka et Al. [6] suggèrent que l’étiopathogénie de la SVN serait inflammatoire dans les formes localisées et néoplasique dans les formes diffuses. Pour notre patiente, la notion d'entorse à répétition a été retrouvée. La présentation clinique des SVN est aspécifique. Dans les deux formes, l’évolution est lente, généralement le diagnostic se fait à un stade avancé de la maladie, d'environ 2 à 3 ans après le début. Dans notre cas, le délai était de 20 mois. Les principaux signes cliniques de la SVN sont l'apparition d'une tuméfaction mono articulaire parfois palpable sous la forme d'une masse, responsable d'une douleur mécanique ainsi que d'une gêne fonctionnelled'aggravation progressive. La symptomatologie clinique de la SVN est donc non spécifique, sans signe pathognomonique, rendant le diagnostic difficile. Les examens complémentaires revêtent alors une importance capitale dans cette pathologie.

Les radiographies standards sont en général normales. Des érosions osseuses sont parfois observéesà un stade avancé du fait du caractère destructeur de la SVN. Dans les formes localisées, l’échographie peut objectiver une formation hypoéchogène qui peut être associée à un épanchement articulaire. L'IRM reste l'examen de choix et peut montrer de multiples lésions synoviales présentant un signal hypo-intense ou intermédiaire en T1 et un hypo signal enT2 et écho de gradient [7]. Les villosités touchées sont bien distinguées du liquide articulaire. De petites zones d'hypo signal sont mises en évidence dans les masses synoviales sans rehaussement du signal par le gadolinium ce qui correspond à des dépôts d'hémosidérine. Cet aspect à l'IRM est quasi pathognomonique de la SVN. En outre, l'IRM permet de déceler une éventuelle récidive lors de la surveillance de la maladie. Dans notre cas, l'IRM nous a permis d’évoquer fortement le diagnostic, mais ne peut pas remplacer l’étude histologique qui reste indispensable pour confirmer ou affirmer le diagnostic. L’évolution locorégionale de la SVN est lente avec une extension à toute la synoviale, à l'os et aux parties molles adjacentes. Une SVN localisée non traitée peut évoluer vers une forme diffuse, d'où l'intérêt d'un traitement précoce et adéquat. Ce traitement n'est pas encore standardisé, il n'y a pas de stratégie thérapeutique uniforme du fait de la rareté de cette affection. Il repose en premier lieu sur une synovectomie chirurgicale soigneuse, la plus complète possible. Dans les formes localisées, comme notre cas, une synovectomie partielle est la règle, qui peut êtreréalisée sous arthroscopie ou par un abord chirurgical. Dines et al. [8] rapportent 90% de résultats excellents après résection arthroscopique à 5 ans du recul. Cependant Sharma et Al. [9] rapportent un taux de récidiveplus élevé chez les patients ayant bénéficié d'un traitement par arthroscopie (59%) que chez ceux traités par la chirurgie à ciel ouvert (29%). Dans les formes diffuses, la synovectomie totale doit être complétée par un curetage osseux. Certains auteurs préconisentles synoviorthèses (isotopiques ou osmiques) dans les cas où la synovectomie est incomplète. D'autres recommandent l'utilisation d'anti-tumorn ecrosis factor alpha (anti-TNF-α) chez des patients refusant les traitements habituels, mais son efficacité est discutée. Le pronostic à long terme de la maladie dépend de l'extension de la lésion au moment du diagnostic, de la localisation et de la qualité de l′exérèse. Généralement les formes localisées guérissent après exérèse complète, avec un risque de récidive quasi nul [10]. Pour notre patiente, à plus de deux ans de recul, il n'y avait pas de récidive locale.

## Conclusion

La SVN dans sa forme localisée touche avec prédilection le genou (75% des cas), la hanche et rarement la cheville. Le tableau clinique est le plus souvent aspécifique. L'IRM reste l'examen clé dans cette pathologie aussi bien pour le diagnostic, que pour le suivi. Le diagnostic final est posé par l’étude histologique de biopsie synoviale ou de pièce opératoire.
